# Disproportionality analysis of adverse events associated with pacritinib: a real-world study based on FDA Adverse Event Reporting System (FAERS) database

**DOI:** 10.3389/fonc.2025.1618267

**Published:** 2025-07-28

**Authors:** Huiling Zhang, Yaping Huang, Chengjie Ke, Maohua Chen

**Affiliations:** ^1^ Department of Pharmacy, The First People’s Hospital of Nanning, Nanning, China; ^2^ Department of Pharmacy, Shengli Clinical Medical College of Fujian Medical University, Fujian Provincial Hospital, Fuzhou University Affiliated Provincial Hospital, Fuzhou, Fujian, China; ^3^ Department of Pharmacy, The First Affiliated Hospital, Fujian Medical University, Fuzhou, China; ^4^ Department of Pharmacy, National Regional Medical Center, Binhai Campus of the First Affiliated Hospital, Fujian Medical University, Fuzhou, China; ^5^ Department of Pharmacy, Pingtan Comprehensive Experimental Area Hospital, Fuzhou, China

**Keywords:** pacritinib, adverse event, disproportionality analysis, FAERS, real-world study

## Abstract

**Introduction:**

Pacritinib, a selective Janus kinase (JAK) inhibitor, is approved for the treatment of myelofibrosis in adults with severe thrombocytopenia. However, its safety profile in real-world populations remains unclear. The aim of study is provided a comprehensive profile of pacritinib's safety by evaluating the adverse events (AEs) using a real-world pharmacovigilance database.

**Methods:**

Data from the FDA Adverse Event Reporting System (FAERS) database, spanning from the first quarter of 2022 to the second quarter of 2024, served as the basis for this analysis. To identify potential AE risk signals, several disproportionality analysis methods were applied, including the reporting odds ratio, the proportional reporting ratio, the multi-item gamma Poisson shrinker, and the Bayesian confidence propagation neural network.

**Results:**

A total of 4,304,335 AE reports were collected from the FAERS, with 1,940 reports identifying pacritinib as the primary suspect drug. Significant disproportionality was observed in the following system organ classes: gastrointestinal disorders, investigations, and surgical and medical procedures. Common preferred terms were identified, including diarrhea, fatigue, death, nausea, platelet count decreased, and hemoglobin decreased. Notably, 26 off­-label AEs were also identified.

**Discussion:**

Our study would provide valuable insights for the post-marketing safety surveillance and assessment of pacritinib, and guide its clinical practice.

## 1 Introduction

Myelofibrosis (MF), a type of myeloproliferative neoplasm (MPN), encompasses primary myelofibrosis and secondary myelofibrosis (1). Clinically, MF is characterized by splenomegaly, constitutional symptoms, anemia, thrombocytosis, or thrombocytopenia (2). It is a rare disease, with an incidence of 4 to 6 cases per 100,000 individuals in the United States (3). While conventional treatments can improve or alleviate the clinical symptoms of MF, they fall short of delivering a definitive cure. Allogeneic stem cell transplantation is a potentially curative treatment for MF, but it is hindered by limitations and a low success rate. In recent years, the discovery of pathogenic genes in MPNs has spurred the development of Janus kinase (JAK) inhibitors, offering a novel therapeutic avenue for MF. JAK is a family of non-receptor protein-tyrosine kinases, comprising JAK1, JAK2, JAK3, and tyrosine kinases-2, among which JAK2 mutation (JAK2V617F) plays a pivotal role in the pathogenesis of MPNs (4, 5). JAK inhibitors, specifically targeting JAK2, have demonstrated efficacy in reducing spleen size and alleviating disease-related symptoms (6), thus providing a promising treatment strategy for patients with MF.

Pacritinib is an oral JAK2/FLT3 inhibitor that was approved by the U.S. Food and Drug Administration (FDA) on February 28, 2022, for the treatment of intermediate or high-risk primary or secondary (post-polycythemia vera or post-essential thrombocythemia) myelofibrosis with platelet counts below 50×10^^9^/L (7). Studies have indicated that pacritinib significantly alleviates clinical symptoms in MF patients with cytopenias, particularly by reducing spleen size, thereby improving patients’ quality of life and extending survival (8). However, several adverse events (AEs) have been reported during pacritinib administration, including diarrhea, nausea, vomiting, thrombocytopenia, anemia, peripheral edema, dizziness, and fever, with the potential for severe AEs to occur (9, 10). With the widespread use of pacritinib, it is necessary to conduct post-marketing surveillance and evaluation.

The FDA Adverse Event Reporting System (FAERS) database serves as a spontaneous reporting system for the collection of AE information and medication error information worldwide, covering millions of drug AE reports. It provides support for the FDA’s post-marketing drugs monitor and therapeutic products. This system plays an essential role in identifying and evaluating potential signals while quantifying the associations between specific drugs and AEs reported by individuals. The database is regularly updated every quarter, encompassing a wide range of AE records, documentation of medication errors, and instances of product quality complaints (11, 12). The aim of study is perform signal detection of pacritinib-related AEs in the FAERS database, evaluating the real-world incidence of AEs, identifying new and serious AE signals, and providing reference for the long-term application management of pacritinib.

## 2 Methods

The methods section was based on our previous study (13).

### 2.1 Data source

This pharmacovigilance investigation evaluated the AEs associated with pacritinib, as documented in the FAERS database. Data entries made between the first quarter of 2022 and the second quarter of 2024 were included in the analysis to coincide with the timing of FDA’s approval of pacritinib. The FAERS data were obtained from the official FDA website, accessed at https://fis.fda.gov/extensions/FPD-QDE-FAERS/FPD-QDE-FAERS.html.

The FAERS data package can be downloaded from the website as a compressed file, comprising seven distinct sections that encompass demographic and management details, drug-related information, AEs, patient outcomes, sources of reporting, treatment duration with reported drugs, indications, and removed cases (14). The intricate process of data manipulation was proficiently executed through the utilization of Python 3.10 (Python Software Foundation, Holland) and Microsoft Excel 2019, collectively contributing to the meticulous data processing endeavors. Fuzzy matching techniques were utilized to filter reports referencing the drug’s generic name, pacritinib, and its product name, Vonjo^®^, specifically tagged as “primary suspect” drug names in the database. Given that this study hinged on a globally accessible database containing de-identified information without direct treatment intervention or the collection of human samples, the requirement for informed consent was deemed unnecessary.

### 2.2 Data extraction

**Figure 1:**
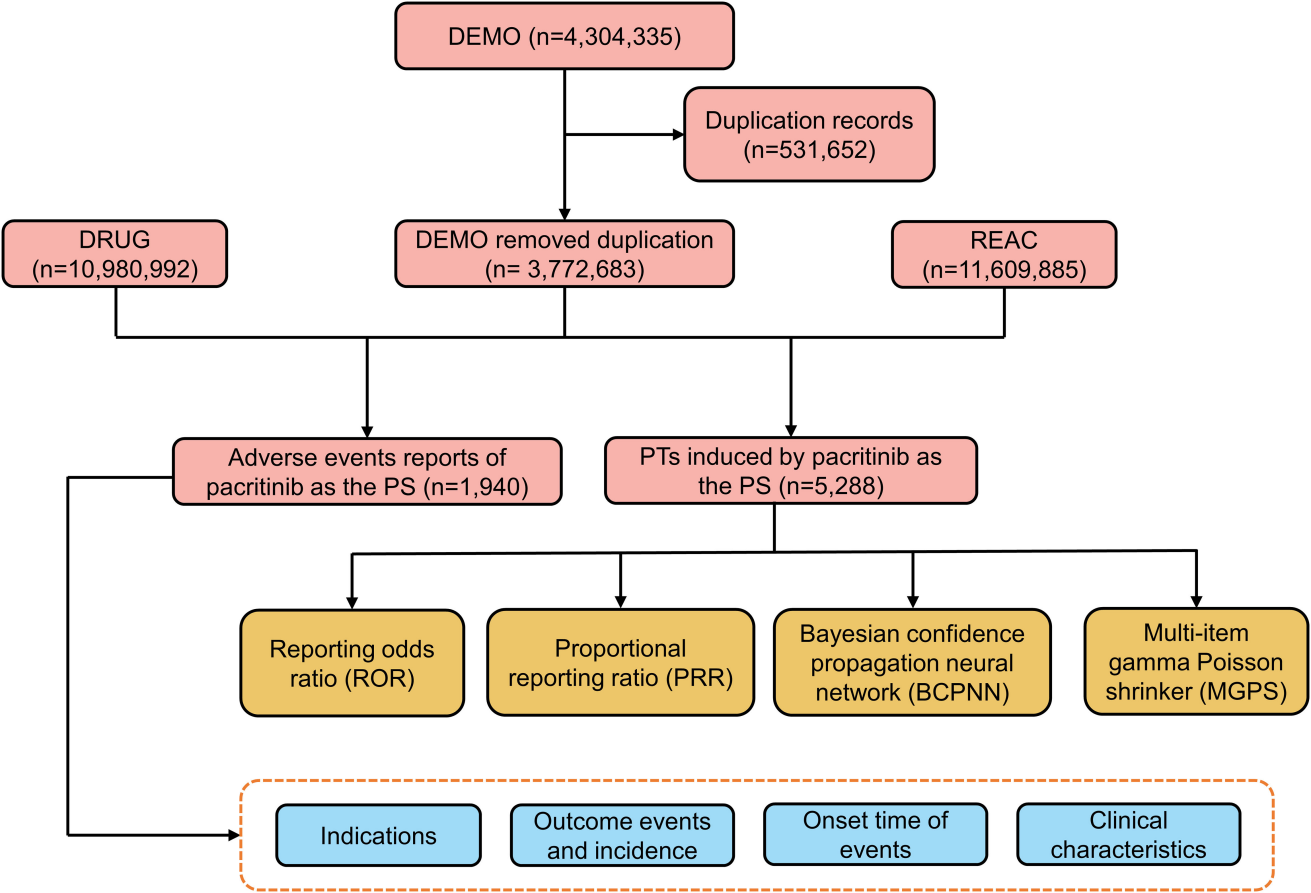
The flowchart of the data analysis.

Due to the inherent spontaneity of the reports, duplication was likely to occur. Therefore, a deduplication process was performed before the analysis, per the FDA guidelines (15). We manually reviewed the reports to exclude instances where the PRIMARYID was lower when the CASEID matched. Additionally, we excluded any CASEID listed in the deleted cases file. AEs in the FAERS are classified based on the preferred terms (PTs) from the standardized Medical Dictionary for Regulatory Activities 26.0 (MedDRA 26.0). The classification is made into five levels: system organ class (SOC), high-level group term (HLGT), high-level term (HLT), PT, and lowest-level term (LLT) (15, 16). **Figure 1** depicts a flowchart to illustrate the sequential procedures involved in extracting, processing, and analyzing data.

In the context of AEs in drug-related reports, it is the responsibility of the individuals reporting these events to assign appropriate codes. The available codes for assignment were: 1 = suspect, 2 = concomitant, and 3 = interacting. To ensure utmost precision and accuracy in our analysis, we made a strategic decision to designate code “1” as “PS” (primary suspected) within the DRUG files with the aim of enhancing analytical accuracy and overall conclusions (11).

### 2.3 Data mining

Disproportionality analysis is a vital technique employed in pharmacovigilance studies, playing a pivotal role in the identification of potential signals indicating AEs associated with a drug. This approach entails comparing the frequency of AEs linked to a specific medication against the occurrence of AEs related to all other drugs. Essentially, it relies on the concept that a signal emerges during data extraction when the incidence rate of a particular AE for a given drug significantly surpasses the background occurrence rate observed across the entire database. This deviation from normality must exceed a predetermined threshold or set of criteria to be considered statistically significant (11). To explore the association between pacritinib and AEs, we applied both Bayesian and frequentist approaches, employing various statistical measures such as the reporting odds ratio (ROR), proportional reporting ratio (PRR), information component (IC), and empirical Bayes geometric mean (EBGM) (17–19). The equations and criteria for these four algorithms are detailed in **Supplementary Tables S1** and **S2**.

## 3 Results

### 3.1 Descriptive analysis

A total of 4,304,335 AE reports were collected from the FAERS database from Q1 2022 to Q2 2024. After removing duplicate reports, 1,940 reports of pacritinib as the PS drug were obtained (**Figure 1**). The clinical characteristics of AEs associated with pacritinib were summarized in **Table 1**. Among the recorded AEs, the proportion of males (56.18%) was slightly higher than that of females (43.82%); most of the patients who experienced AEs were aged 65 years or older (72.78%), with a median age of approximately 72 years (range: 65-77 years). A total of 634 (32.68%) serious AEs were reported, including 335 (52.84%) cases of hospitalization and 220 (34.70%) cases of death. The majority of these AE reports originated from the United States (99.07%), and most were reported by consumers (84.78%). Since the drug’s market introduction, the highest number of AEs was reported in 2023 (55.31%).

**Table 1:** The clinical characteristics of AEs associated with pacritinib.

Characteristics	Pacritinib-induced AE reports (n = 1940)	
	Case number (n)	Case proportion (%)
Gender	356	18.35%
Female	156	43.82%
Male	200	56.18%
Age (years)	338	17.42%
< 18	0	0.00%
18 ≤ and ≤ 65	92	27.22%
> 65	246	72.78%
Median (IQR)	72 (65-77)	–
Reported countries	1940	100.00%
United States	1922	99.07%
Poland	4	0.21%
Other country	14	0.73%
Reporters	1925	99.23%
Health professional	293	15.22%
Consumer	1632	84.78%
Reporting year	1940	100.00%
2022	375	19.33%
2023	1073	55.31%
2024 (Q1-Q2)	492	25.36%
Outcomes	1940	100.00%
Non-serious	1306	67.32%
Serious	634	32.68%
Death	220	34.70%
Disability	0	0.00%
Life-threatening	2	0.32%
Hospitalization	335	52.84%
Other Serious Outcome	185	29.18%
Time-to-onset (days)	470	24.23%
Median (IQR)	27 (2-115.75)	–

AEs, adverse events; IQR, interquartile range; Q, quarter.

### 3.2 Disproportionality analysis

The AEs associated with pacritinib detected in this study mainly involved 24 SOCs. The disproportionality analysis signals at the SOC level were described in **Table 2**. The SOCs meeting all four algorithm criteria were gastrointestinal disorders (n=659, ROR 2.62, PRR 2.36, IC 1.24, EBGM 2.36), investigations (n=485, ROR 2.29, PRR 2.14, IC 1.10, EBGM 2.14), and surgical and medical procedures (n=188, ROR 2.18, PRR 2.13, IC 1.09, EBGM 2.13). Additionally, SOCs meeting one of the four algorithm criteria included general disorders and administration site conditions (n=811, ROR 1.11, IC 0.12), blood and lymphatic system disorders (n=125, ROR 1.50, IC 0.57), metabolism and nutrition disorders (n=105, ROR 1.12, IC 0.16), and social circumstances (n=47, ROR 1.86, IC 0.89). Of note, some signals unrelated to drug AEs were also collected in this study, including injury, poisoning and procedural complications, surgical and medical procedures, and social circumstances.

**Table 2:** Signal strength of AEs of pacritinib at the SOC level.

SOC	Number	ROR (95% CI)	PRR (χ^2^)	IC (IC025)	EBGM (EBGM05)
General disorders and administration site conditions	811	1.11 (1.03-1.20)	1.09 (7.40)	0.12 (0.12)	1.09 (1.01)
Gastrointestinal disorders	659	2.62 (2.41-2.84)	2.36 (554.70)	1.24 (1.14)	2.36 (2.17)
Injury, poisoning and procedural complications	542	0.95 (0.87-1.04)	0.95 (1.40)	-0.07 (-0.07)	0.95 (0.87)
Investigations	485	2.29 (2.08-2.52)	2.14 (311.31)	1.10 (1.00)	2.14 (1.95)
Nervous system disorders	235	0.80 (0.70-0.91)	0.81 (11.44)	-0.31 (-0.35)	0.81 (0.71)
Infections and infestations	194	0.73 (0.64-0.85)	0.75 (17.78)	-0.42 (-0.49)	0.75 (0.65)
Surgical and medical procedures	188	2.18 (1.88-2.52)	2.13 (114.47)	1.09 (0.94)	2.13 (1.84)
Skin and subcutaneous tissue disorders	147	0.72 (0.61-0.85)	0.73 (15.02)	-0.45 (-0.53)	0.73 (0.62)
Respiratory, thoracic and mediastinal disorders	144	0.81 (0.69-0.96)	0.82 (6.18)	-0.29 (-0.34)	0.82 (0.69)
Psychiatric disorders	138	0.68 (0.58-0.81)	0.69 (19.78)	-0.53 (-0.63)	0.69 (0.58)
Blood and lymphatic system disorders	125	1.50 (1.26-1.80)	1.49 (20.40)	0.57 (0.48)	1.49 (1.25)
Metabolism and nutrition disorders	105	1.12 (0.93-1.36)	1.12 (1.40)	0.16 (0.14)	1.12 (0.92)
Musculoskeletal and connective tissue disorders	100	0.53 (0.44-0.65)	0.54 (40.34)	-0.88 (-1.08)	0.54 (0.44)
Cardiac disorders	66	0.75 (0.58-0.95)	0.75 (5.62)	-0.42 (-0.53)	0.75 (0.59)
Vascular disorders	60	0.61 (0.47-0.78)	0.61 (14.99)	-0.71 (-0.91)	0.61 (0.48)
Social circumstances	47	1.86 (1.40-2.48)	1.85 (18.51)	0.89 (0.67)	1.85 (1.39)
Renal and urinary disorders	46	0.61 (0.46-0.82)	0.61 (11.33)	-0.70 (-0.94)	0.61 (0.46)
Neoplasms benign, malignant and unspecified (incl cysts and polyps)	34	0.21 (0.15-0.29)	0.22 (100.64)	-2.21 (-3.10)	0.22 (0.15)
Eye disorders	23	0.29 (0.19-0.43)	0.29 (40.74)	-1.79 (-2.69)	0.29 (0.19)
Immune system disorders	20	0.32 (0.21-0.50)	0.33 (28.42)	-1.62 (-2.51)	0.33 (0.21)
Hepatobiliary disorders	11	0.26 (0.15-0.47)	0.26 (22.70)	-1.92 (-3.47)	0.26 (0.15)
Reproductive system and breast disorders	10	0.37 (0.20-0.68)	0.37 (10.90)	-1.44 (-2.68)	0.37 (0.20)
Ear and labyrinth disorders	8	0.37 (0.18-0.74)	0.37 (8.63)	-1.43 (-2.87)	0.37 (0.18)
Product issues	8	0.08 (0.04-0.16)	0.08 (84.28)	-3.61 (-7.22)	0.08 (0.04)

SOC, system organ classes; CI, confidence interval; ROR, reporting odds ratio; PRR, proportional reporting ratio; χ^2^, chi-squared; IC, information component; IC025, the lower limit of the 95 two-sided CI of the IC; EBGM, empirical bayesian geometric mean; EBGM05, the lower 95 two-sided CI of EBGM.

A total of 60 significant disproportionality PTs related to pacritinib were detected across 14 SOCs by applying all four algorithms in combination. The top 10 most common reports were diarrhea (n=374), fatigue (n=223), death (n=194), nausea (n=183), platelet count decreased (n=162), hemoglobin decreased (n=119), asthenia (n=82), splenomegaly (n=61), decreased appetite (n=53), and constipation (n=52). **Table 3** presents the signal strength of AEs listed in the pacritinib label. Among these, diarrhea, nausea, platelet count decreased, hemoglobin decreased, and peripheral swelling were consistent with the common AEs enumerated in the drug label. Notably, after comparing with the drug label, our study identified 26 PTs not listed in the label (**Table 4**), such as asthenia (n=82), decreased appetite (n=53), constipation (n=52), abdominal discomfort (n=45), night sweats (n=18), pulmonary oedema (n=12), which may represent new potential AEs of pacritinib. Furthermore, **Supplementary Table S4** presents PTs that are unrelated to AEs of pacritinib.

**Table 3:** The signal strength of AEs of pacritinib at the PT level(label-listed AEs).

SOC	PTs	Number	ROR (95% CI)	PRR (χ^2^)	IC (IC025)	EBGM (EBGM05)
Gastrointestinal disorders	Diarrhoea	374	7.18 (6.46-7.98)	6.74 (1843.82)	2.75 (2.48)	6.73 (6.06)
	Nausea	183	3.16 (2.72-3.66)	3.08 (260.18)	1.62 (1.40)	3.08 (2.66)
	Mouth haemorrhage	5	9.45 (3.92-22.76)	9.44 (37.58)	3.23 (1.34)	9.41 (3.91)
General disorders and administration site conditions	Fatigue	223	3.26 (2.85-3.73)	3.17 (334.59)	1.66 (1.45)	3.16 (2.77)
	Peripheral swelling	46	2.93 (2.19-3.91)	2.91 (57.77)	1.54 (1.15)	2.91 (2.17)
	Localised oedema	3	10.78 (3.47-33.53)	10.77 (26.47)	3.42 (1.10)	10.73 (3.45)
Investigations	Platelet count decreased	162	17.33 (14.81-20.27)	16.83 (2397.35)	4.06 (3.47)	16.70 (14.28)
	Red blood cell count decreased	27	9.85 (6.74-14.39)	9.80 (212.59)	3.29 (2.25)	9.76 (6.68)
	Haemoglobin decreased	119	15.92 (13.27-19.11)	15.59 (1615.46)	3.95 (3.29)	15.48 (12.90)
Metabolism and nutrition disorders	Fluid retention	14	3.88 (2.30-6.57)	3.88 (29.85)	1.95 (1.16)	3.87 (2.29)
Respiratory, thoracic and mediastinal disorders	Epistaxis	28	5.61 (3.87-8.13)	5.58 (105.17)	2.48 (1.71)	5.57 (3.84)
Skin and subcutaneous tissue disorders	Blood blister	4	18.81 (7.03-50.34)	18.80 (66.82)	4.22 (1.58)	18.64 (6.97)
Eye disorders	Conjunctival haemorrhage	3	12.55 (4.03-39.04)	12.54 (31.68)	3.64 (1.17)	12.47 (4.01)
Vascular disorders	Haemorrhage	25	3.15 (2.12-4.66)	3.14 (36.36)	1.65 (1.11)	3.13 (2.11)

SOC, system organ classes; PT, preferred term; CI, confidence interval; ROR, reporting odds ratio; PRR, proportional reporting ratio; χ^2^, chi-squared; IC, information component; IC025, the lower limit of the 95 two-sided CI of the IC; EBGM, empirical bayesian geometric mean; EBGM05, the lower 95 two-sided CI of EBGM.

**Table 4:** The signal strength of AEs of pacritinib at the PT level( AEs not stated in the labeling).

SOC	PTs	Number	ROR (95% CI)	PRR (χ^2^)	IC (IC025)	EBGM (EBGM05)
Gastrointestinal disorders	Constipation^a^	52	2.87 (2.18-3.77)	2.85 (62.48)	1.51 (1.15)	2.85 (2.16)
	Abdominal discomfort^a^	45	2.94 (2.19-3.95)	2.93 (57.12)	1.55 (1.15)	2.92 (2.18)
	Faeces soft^a^	6	5.85 (2.62-13.03)	5.84 (24.01)	2.54 (1.14)	5.83 (2.61)
	Bowel movement irregularity^a^	6	7.04 (3.16-15.69)	7.03 (30.94)	2.81 (1.26)	7.01 (3.14)
	Oral mucosal blistering^a^	3	6.24 (2.01-19.38)	6.23 (13.15)	2.64 (0.85)	6.22 (2.00)
General disorders and administration site conditions	Asthenia^a^	82	2.86 (2.30-3.55)	2.83 (97.31)	1.50 (1.20)	2.83 (2.27)
	Energy increased^b^	12	30.44 (17.21-53.84)	30.37 (336.22)	4.91 (2.77)	29.97 (16.94)
Investigations	Full blood count decreased^b^	19	9.02 (5.75-14.17)	9.00 (134.52)	3.16 (2.01)	8.96 (5.71)
	Renal function test abnormal^b^	4	8.51 (3.19-22.73)	8.51 (26.39)	3.08 (1.15)	8.48 (3.17)
	Full blood count abnormal^b^	28	9.65 (6.65-14.00)	9.60 (214.98)	3.26 (2.25)	9.57 (6.59)
	Platelet count increased^b^	43	34.16 (25.25-46.23)	33.89 (1352.09)	5.06 (3.74)	33.39 (24.68)
	Platelet count abnormal^b^	12	19.72 (11.17-34.84)	19.68 (210.90)	4.29 (2.43)	19.51 (11.05)
	Haemoglobin increased^b^	16	40.38 (24.61-66.26)	40.26 (601.61)	5.31 (3.23)	39.56 (24.11)
	Haemoglobin abnormal^b^	14	24.52 (14.47-41.55)	24.46 (311.56)	4.60 (2.71)	24.20 (14.28)
	Haematocrit decreased^b^	10	8.31 (4.46-15.47)	8.29 (63.91)	3.05 (1.64)	8.27 (4.44)
	Blood uric acid increased^a^	4	7.70 (2.89-20.57)	7.70 (23.24)	2.94 (1.10)	7.68 (2.87)
	Laboratory test abnormal^b^	22	9.15 (6.02-13.93)	9.12 (158.48)	3.18 (2.09)	9.09 (5.97)
	Full blood count increased^b^	7	67.16 (31.65-142.54)	67.08 (442.13)	6.02 (2.84)	65.12 (30.68)
	White blood cell count increased^b^	18	7.11 (4.47-11.30)	7.09 (93.83)	2.82 (1.77)	7.07 (4.45)
	White blood cell count normal^b^	3	3293.37 (550.17-19714.23)	3291.50 (3947.40)	10.36 (1.73)	1317.20 (220.05)
Metabolism and nutrition disorders	Decreased appetite^a^	53	2.64 (2.02-3.46)	2.63 (53.52)	1.39 (1.06)	2.62 (2.00)
	Gout^a^	9	6.30 (3.27-12.13)	6.29 (39.95)	2.65 (1.38)	6.28 (3.26)
	Fluid intake reduced^b^	3	7.24 (2.33-22.49)	7.23 (16.07)	2.85 (0.92)	7.21 (2.32)
Respiratory, thoracic and mediastinal disorders	Pulmonary oedema^a^	12	3.90 (2.21-6.88)	3.89 (25.79)	1.96 (1.11)	3.89 (2.21)
Skin and subcutaneous tissue disorders	Night sweats^a^	18	6.91 (4.35-10.99)	6.89 (90.43)	2.78 (1.75)	6.87 (4.32)
Nervous system disorders	Ageusia^a^	9	4.47 (2.32-8.61)	4.47 (24.18)	2.16 (1.12)	4.46 (2.32)

SOC,system organ classes; PT, preferred term; CI, confidence interval; ROR, reporting odds ratio; PRR, proportional reporting ratio; χ^2^, chi-squared; IC, information component; IC025, the lower limit of the 95 two-sided CI of the IC; EBGM, empirical bayesian geometric mean; EBGM05, the lower 95 two-sided CI of EBGM.

a Biologically plausible but under-powered observations.

b Probable artefacts (e.g., laboratory codes).

### 3.3 Onset time of events

**Figure 2:**
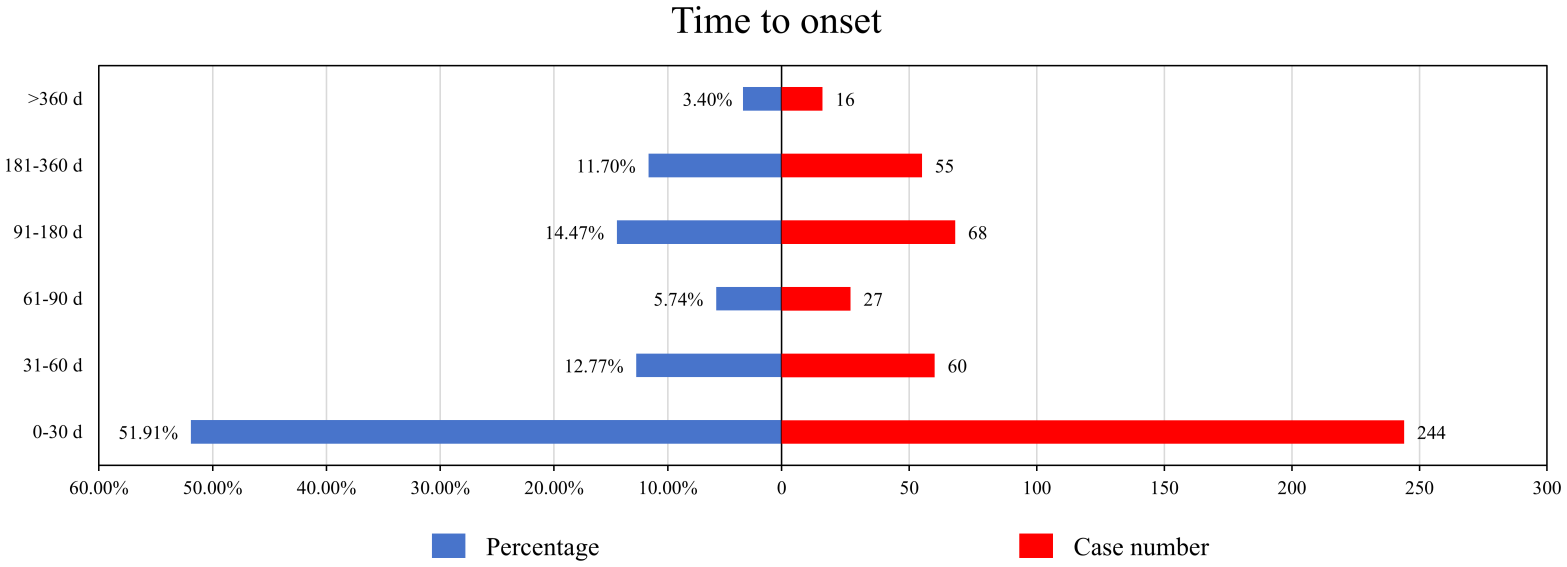
Time to onset of pacritinib-related AEs.

After excluding reports with missing data and duplicates, a total of 470 AEs included the time to onset. The median onset time for pacritinib-related AEs was 27 days (interquartile range 2-115.75 days). As shown in **Figure 2**, more than half of the AEs occurred within the first month (n=244, 51.91%) after starting pacritinib treatment. The frequency of AEs decreased over time, but there was a resurgence in AEs during the 3 to 12-month period (n=123, 26.17%) after initiation. Remarkably, even after one year of pacritinib treatment, 3.40% (n=16) of the AEs were still reported.

## 4 Discussion

Current studies have indicated that the pathogenesis of MF is frequently linked to dysregulation of the JAK2 signaling pathway. Specifically, the V617F mutation in the JH2 domain of JAK2 causes a loss of the negative regulatory function of JH1, resulting in excessive collagen proliferation in the bone marrow hematopoietic tissue, thereby triggering MF (20). The safety of pacritinib, a novel JAK inhibitor used for the treatment of MF with severe thrombocytopenia, raises critical concern for healthcare providers and patients. Our study provides the AEs profile of pacritinib in real-world settings, which would help clinicians in making more comprehensive and accurate treatment decisions during the treatment of pacritinib in MF.

Our study revealed a male-to-female ratio of 1.28:1 in pacritinib-related AE reports, with the majority of events involving individuals aged 65 or older. This finding is consistent with the epidemiological characteristics of MF, which exhibits a slightly higher incidence in males, and a median age of initial diagnosis at 65 years (21, 22). However, the substantial proportion of missing data on gender (81.65%) and age (82.58%) may affect these results. Notably, 99.07% of AE reporting country is the United States, a consequence of pacritinib’s lack of approval outside North America. Therefore, the potential differences in disease incidence and AE rates among different ethnic groups cannot be excluded.

The most frequently reported AEs associated with pacritinib in our findings were gastrointestinal disorders, notably diarrhea (n=374), nausea (n=183), and constipation (n=52), which was consistent with earlier studies. Previous phase II clinical trials indicated that the most common AEs in patients receiving pacritinib were grade 1 and 2 diarrhea, nausea, and vomiting (23, 24). Similarly, the most common AEs in the pacritinib group were observed in a larger randomized, controlled, phase III trials (PERSIST-1), including diarrhea (n=120, 55%), nausea (n=60, 27%), and vomiting (n=36, 16%), and 9 (4%) patients discontinued treatment due to gastrointestinal AEs (25). Diarrhea emerged as the most frequent gastrointestinal AE associated with pacritinib and was generally manageable with standard antidiarrheal medications, typically resolving within 1 to 2 weeks (9). Current evidence suggests that pacritinib-related gastrointestinal AEs may be related to FLT3 inhibition (6), with most events being grade 1 or 2 and within manageable limits. However, severe gastrointestinal events, such as diarrhea, warrant still be monitored closely.

Hematologic toxicity is a common AE of JAK inhibitors, often manifesting as febrile neutropenia, anemia, and thrombocytopenia (26). Pacritinib, which selectively inhibits JAK2, FLT3, c-fms, and IRAK1, but not inhibit JAK1 (27), is associated with less bone marrow suppression compared to other JAK inhibitors such as ruxolitinib and fedratinib. This reduced bone marrow suppression is likely due to the selective inhibition of JAK2 while sparing JAK1, and the reduction of hematopoietic suppressive cytokines through inhibition of interleukin-1 receptor-associated kinase 1 and colony-stimulating factor 1 receptor (28). Thus, pacritinib is a safer option for MF patients with severe thrombocytopenia. A phase III trials observed hematologic AEs including anemia and thrombocytopenia (9, 25), and a retrospective analysis of safety data from 189 patients in a phase III trial showed that the most common grade 3 or 4 AEs were thrombocytopenia (34.8%) and anemia (31.8%), though these rarely necessitated dose reductions or discontinuation (29). Our study also identified a substantial number of reports related to thrombocytopenia and hemoglobin decreased, but the detailed information on the severity and correlation of these AEs were lacked. Additionally, our results indicated strong signal strength for hematologic-related investigations, such as hemoglobin increased (ROR 40.38, PRR 40.26, IC 5.31, EBGM 39.56) and platelet count increased (ROR 34.16, PRR 33.89, IC 5.06, EBGM 33.39), suggesting that necessary for intensive monitoring of hematologic parameters during pacritinib treatment.

The PERSIST-1 trial identified the most common severe AEs associated with pacritinib as grade 3 or 4 bleeding (3%) and cardiac events (8%) (25). Similarly, the multicenter randomized phase III study (PERSIST-2) reported grade 3 or 4 bleeding (10.5%) and cardiac events (9.5%), rates comparable to those observed in the Best Available Therapy (BAT) group (9). Risk-adjusted analyses of early-phase clinical trials have demonstrated that the incidence of fatal AEs with pacritinib is lower than that associated with BAT, with no increased occurrences of bleeding, cardiac events, secondary malignancies, or thrombosis (30). Concerns regarding these severe AEs have prompted adjustments in patient exclusion criteria in subsequent dose-exploration PAC203 clinical trials. Patients receiving pacritinib did not experience grade 3 or higher bleeding or cardiac events (31). In our study, while we did not observe AEs such as cardiac failure or prolonged QT interval, we did identify bleeding-related AEs, including hemorrhage, conjunctival hemorrhage, epistaxis, mouth hemorrhage, and blood blister. Although severe AEs like intracerebral hemorrhage or meningeal bleeding were not reported, it is important to monitor and assess these potential AEs during pacritinib treatment in MF patients.

In this study, we not only identified common AEs labeled in pacritinib’s package insert, including diarrhea, thrombocytopenia, nausea, peripheral edema, and hemorrhage, but also discovered 26 potential AEs not listed in the label. Among them, constipation (n=52), abdominal discomfort (n=45), decreased appetite (n=53), and asthenia (n=82) are of significant clinical concern. Literature comparison analysis shows that these newly identified AEs have been clearly reported in similar JAK2/FLT3 inhibitors: phase III trial data of fedratinib indicates that 27% of patients experienced constipation and 24% asthenia (32); in ruxolitinib treatment, 5.5% of patients had constipation, 3.2% had decreased appetite, and 15.4% presented with asthenia (33). Such consistency suggests that these AEs may be associated with the pharmacological mechanisms of JAK inhibitors. It is noteworthy that constipation, decreased appetite, and abdominal discomfort have been documented in the phase I/II studies of pacritinib, and this study further confirms their clinical relevance by expanding the sample size. Future research should combine pharmacogenomic analysis to explore their molecular mechanisms.

Our study also detected some AE signals associated with primary diseases, such as splenomegaly and splenic disorders. Additionally, a total of 194 death reports were collected in this study (ROR 2.75, PRR 2.69, IC 1.43, EBGM 2.69). Given that this study cannot exclude the influence of primary disease, concomitant medications, dosage, and treatment duration, the findings only suggest a statistical association between the drug and AEs. The determination of causality between pacritinib and these events must be assessed in the context of evidence-based medicine. It is emphasized that studies have shown pacritinib may have a lower risk of death compared to the best available therapy (BAT) (hazard ratio [HR] = 0.68, 95% CI: 0.30–1.53) (25). Based on this evidence, the “death” signals identified in FAERS may partially reflect progression of patients’ underlying diseases (e.g., myelofibrosis) rather than direct drug toxicity.

In addition to potential disease progression or comorbidities, concomitant medications are a significant confounding factor in attributing AEs. Patients in the study commonly used multiple concomitant medications, whose potential drug interactions may significantly impact the safety profile of pacritinib. Pacritinib is primarily metabolized by the cytochrome P450 3A (CYP3A) enzyme system, making it susceptible to pharmacokinetic influences from CYP3A inducers or inhibitors (4). For example, co-administration with potent CYP3A inhibitors (such as itraconazole, ketoconazole, voriconazole, clarithromycin) can significantly increase the concentration curve of pacritinib, resulting in elevated plasma drug concentrations (34). This may consequently increase the risk of serious AEs such as myelosuppression and hemorrhage. Pacritinib has been shown *in vitro* to be an inhibitor of P-glycoprotein (P-gp), breast cancer resistance protein (BCRP), and organic cation transporter 1 (OCT1). When co-administered with substrates of P-gp, BCRP, or OCT1, pacritinib may increase the plasma concentrations of these substrates by inhibiting transporter activity, potentially leading to serious adverse events (35).

However, this study acknowledges several limitations inherent in using data from the FAERS spontaneous reporting system. First, as a spontaneous reporting system, FAERS may have cases of underreporting or duplicate reporting of adverse events. Second, as a newly approved drug, pacritinib tends to receive heightened attention from the media and clinicians, which may lead to an increase in the number of AE reports associated with it. Third, the data analyzed in this study show that a substantial number of AE reports originate from consumers, and the information provided in such reports may be inaccurate or incomplete, potentially introducing biases into the data. Fourth, the occurrence of AEs described in the reports may be influenced by multiple factors such as progression of the underlying disease, concomitant diseases and medications, off-label use, and drug interactions, rather than being directly caused by pacritinib. Meanwhile, insufficient reporting information limits comprehensive causal assessment, making it impossible to clearly attribute the AEs in the FAERS database to pacritinib. Finally, the FAERS database does not include all AE reports related to pacritinib, so the true incidence of pacritinib-related AEs in the medication population cannot be calculated. Despite the above-mentioned multiple limitations, this study enhances the reliability of the identified potential AEs through cross-validation of four data mining algorithms, providing valuable supplementary evidence for the safety monitoring of pacritinib.

## 5 Conclusion

Using the FAERS database, a comprehensive analysis concerning pacritinib and AEs among MF patients was conducted. Several common AEs that align with those listed in the prescribing information were identified, such as diarrhea, vomiting, nausea, anemia, and thrombocytopenia. Moreover, 26 new AE signals not included in the prescribing information were detected. These findings highlight the necessity of continuing monitoring and further investigation into these emerging risk signals. Overall, our research provides valuable insights for the post-marketing safety surveillance and assessment of pacritinib.

## Data Availability

The original contributions presented in the study are included in the article/**Supplementary Material**. Further inquiries can be directed to the corresponding authors.

## Supplementary material

Supplementary Table 1 Two-by-two contingency table for disproportionality analyses. Equation: a, number of reports containing both the target drug and target adverse drug reaction; b, number of reports containing other adverse drug reaction of the target drug; c, number of reports containing the target adverse drug reaction of other drugs; d, number of reports containing other drugs and other adverse drug reactions.

Supplementary Table 2 Summary of major algorithms used for signal detection. N, number of adverse event reports; CI, confidence interval; ROR, reporting odds ratio; ROR05, the lower limit of the 95 two-sided CI of the ROR; N, the number of co-occurrences; PRR, proportional reporting ratio; χ^2^, chi-squared; BCPNN, bayesian confidence propagation neural network; IC, information component; IC025, the lower limit of the 95 two-sided CI of the IC; MGPS, multi-item gamma Poisson shrinker; EBGM, empirical bayesian geometric mean; EBGM05, the lower 95 two-sided CI of EBGM.

Supplementary Table 3 The signal strength of PTs associated with diseases *. SOC, system organ classes; PT, preferred terms;ROR, CI, confidence interval; ROR, reporting odds ratio; ROR05, the lower limit of the 95 two-sided CI of the ROR; N, the number of co-occurrences; PRR, proportional reporting ratio; χ^2^, chi-squared; BCPNN, bayesian confidence propagation neural network; IC, information component; IC025, the lower limit of the 95 two-sided CI of the IC; MGPS, multi-item gamma Poisson shrinker; EBGM, empirical bayesian geometric mean; EBGM05, the lower 95 two-sided CI of EBGM. ^*^ PTs associated with the natural disease course, pathophysiological mechanisms, or manifestations of complications.

Supplementary Table 4 The signal strength of AEs of pacritinib-unrelated at the PT level. SOC, system organ classes; PT, preferred terms; ROR, CI, confidence interval; ROR, Abbreviations: reporting odds ratio; ROR05, the lower limit of the 95 two-sided CI of the ROR; N, the number of co-occurrences; PRR, proportional reporting ratio; χ^2^, chi-squared; BCPNN, bayesian confidence propagation neural network; IC, information component; IC025, the lower limit of the 95 two-sided CI of the IC; MGPS, multi-item gamma Poisson shrinker; EBGM, empirical bayesian geometric mean; EBGM05, the lower 95 two-sided CI of EBGM.
