# Supplementary material for: Disproportionality analysis of adverse events associated with pacritinib: a real-world study based on FDA Adverse Event Reporting System (FAERS) database
Source: Front Oncol. 2025 Jul 28;15:1618267. doi: 10.3389/fonc.2025.1618267 (PMC12337178; doi:10.3389/fonc.2025.1618267)
Supplement: Supplementary Table 1 — Two-by-two contingency table for disproportionality analyses. Equation: a, number of reports containing both the target drug and target adverse drug reaction; b, number of reports containing other adverse drug reaction of the target drug; c, number of reports containing the target adverse drug reaction of other drugs; d, number of reports containing other drugs and other adverse drug reactions. [file DataSheet1.docx]

**Table S1** Two-by-two contingency table for disproportionality analyses.

|  | **Target adverse event** | **All other adverse events** | **Total** |
| --- | --- | --- | --- |
| Target drug | a | b | a+b |
| All other drugs | c | d | c+d |
| Total | a+c | b+d | a+b+c+d |

Equation: a, number of reports containing both the target drug and target adverse drug reaction; b, number of reports containing other adverse drug reaction of the target drug; c, number of reports containing the target adverse drug reaction of other drugs; d, number of reports containing other drugs and other adverse drug reactions.

**Table S2** Summary of major algorithms used for signal detection.

| **Algorithms** | **Equation** | **Criteria** |
| --- | --- | --- |
| ROR | ROR = ad/c/b | ROR05 > 1, N ≥ 2 |
|  | 95%CI = e^ln(ROR)±1.96(1/a+1/b+1/c+1/d)^0.5^ |  |
| PRR | PRR = [a/(a+b)]/[c/(c + d)] | PRR≥2 |
|  | χ^2^ = [(ad-bc)^2^ (a+b + c + d)]/[(a+b)(c + d)(a+c)(b + d)] | χ^2^ ≥ 4, N ≥ 3 |
| BCPNN | IC = log2 [a (a+b + c + d)]/[(a+c)(a+b)] | IC025 > 0 |
|  | 95%CI = e^ln(IC)±1.96(1/a+1/b+1/c+1/d)^0.5^ |  |
| MGPS | EBGM = a (a+b + c + d)/(a+c)/(a+b) | EBGM05 > 2, N ≥ 0 |
|  | 95%CI = e^ln(EBGM)±1.96(1/a+1/b+1/c+1/d)^0.5^ |  |

Abbreviations: N, number of adverse event reports; CI, confidence interval; ROR, reporting odds ratio; ROR05, the lower limit of the 95 two-sided CI of the ROR; N, the number of co-occurrences; PRR, proportional reporting ratio; χ^2^, chi-squared; BCPNN, bayesian confidence propagation neural network; IC, information component; IC025, the lower limit of the 95 two-sided CI of the IC; MGPS, multi-item gamma Poisson shrinker; EBGM, empirical bayesian geometric mean; EBGM05, the lower 95 two-sided CI of EBGM.

**Table S3**The signal strength of PTs associated with diseases *.

| **SOC** | **PTs** | **Number** | **ROR (95% CI)** | **PRR (χ^2^)** | **IC (IC025)** | **EBGM (EBGM05)** |
| --- | --- | --- | --- | --- | --- | --- |
| Blood and lymphatic system disorders | Splenomegaly | 61 | 74.31 (57.50-96.05) | 73.47 (4219.67) | 6.15 (4.76) | 71.12 (55.02) |
|  | Spleen disorder | 6 | 46.74 (20.81-104.97) | 46.69 (262.67) | 5.52 (2.46) | 45.74 (20.36) |
| Neoplasms benign, malignant and unspecified (incl cysts and polyps) | Myelofibrosis | 5 | 24.57 (10.17-59.34) | 24.55 (111.68) | 4.60 (1.91) | 24.28 (10.05) |
| General disorders and administration site conditions | Death | 194 | 2.75 (2.39-3.18) | 2.69 (208.36) | 1.43 (1.24) | 2.69 (2.33) |

Abbreviations: SOC, system organ classes; PT, preferred term; CI, confidence interval; ROR, reporting odds ratio; PRR, proportional reporting ratio; χ^2^, chi-squared; IC, information component; IC025, the lower limit of the 95 two-sided CI of the IC; EBGM, empirical bayesian geometric mean; EBGM05, the lower 95 two-sided CI of EBGM.

*PTs associated with the natural disease course, pathophysiological mechanisms, or manifestations of complications.

**Table S4** The signal strength of AEs of pacritinib-unrelated at the PT level.

| **SOC** | **PTs** | **Number** | **ROR (95% CI)** | **PRR (χ^2^)** | **IC (IC025)** | **EBGM (EBGM05)** |
| --- | --- | --- | --- | --- | --- | --- |
| Social circumstances | Loss of personal independence in daily activities | 37 | 5.27 (3.81-7.28) | 5.24 (126.73) | 2.39 (1.73) | 5.23 (3.78) |
| Surgical and medical procedures | Transfusion | 83 | 113.99 (91.28-142.36) | 112.22 (8705.00) | 6.74 (5.40) | 106.81 (85.52) |
|  | Therapy change | 9 | 9.79 (5.08-18.86) | 9.78 (70.61) | 3.28 (1.71) | 9.74 (5.06) |
|  | Hospice care | 26 | 20.44 (13.88-30.10) | 20.34 (473.83) | 4.33 (2.94) | 20.16 (13.69) |
|  | Bone marrow transplant | 6 | 40.18 (17.91-90.14) | 40.14 (224.88) | 5.30 (2.36) | 39.44 (17.58) |
|  | Platelet transfusion | 6 | 44.99 (20.04-101.00) | 44.94 (252.58) | 5.46 (2.43) | 44.05 (19.62) |
|  | Infusion | 4 | 79.13 (29.17-214.65) | 79.08 (297.63) | 6.25 (2.31) | 76.36 (28.15) |
|  | Spleen operation | 3 | 470.48 (135.16-1637.65) | 470.21 (1156.77) | 8.60 (2.47) | 387.41 (111.30) |
|  | Stem cell transplant | 6 | 45.93 (20.45-103.13) | 45.87 (257.99) | 5.49 (2.45) | 44.96 (20.02) |
|  | Radiotherapy | 4 | 23.18 (8.65-62.09) | 23.16 (83.93) | 4.52 (1.69) | 22.93 (8.56) |
|  | Splenectomy | 4 | 67.05 (24.79-181.40) | 67.00 (252.37) | 6.02 (2.23) | 65.05 (24.04) |
| Injury, poisoning and procedural complications | Product dose omission issue | 199 | 3.68 (3.20-4.24) | 3.58 (373.55) | 1.84 (1.60) | 3.58 (3.10) |
|  | Contusion | 30 | 3.88 (2.71-5.56) | 3.87 (63.77) | 1.95 (1.36) | 3.86 (2.70) |
|  | Post procedural haemorrhage | 5 | 7.89 (3.28-18.99) | 7.88 (29.94) | 2.97 (1.24) | 7.86 (3.26) |
|  | Off label use | 252 | 2.36 (2.08-2.68) | 2.30 (188.39) | 1.20 (1.06) | 2.30 (2.02) |
| General disorders and administration site conditions | Adverse eventa | 21 | 3.26 (2.13-5.01) | 3.25 (32.80) | 1.70 (1.11) | 3.25 (2.12) |

Abbreviations: SOC, system organ classes; PT, preferred term; CI, confidence interval; ROR, reporting odds ratio; PRR, proportional reporting ratio; χ^2^, chi-squared; IC, information component; IC025, the lower limit of the 95 two-sided CI of the IC; EBGM, empirical bayesian geometric mean; EBGM05, the lower 95 two-sided CI of EBGM.
